# Blinding in randomized controlled trials in general and abdominal surgery: protocol for a systematic review and empirical study

**DOI:** 10.1186/s13643-016-0226-4

**Published:** 2016-03-24

**Authors:** Pascal Probst, Kathrin Grummich, Patrick Heger, Steffen Zaschke, Phillip Knebel, Alexis Ulrich, Markus W. Büchler, Markus K. Diener

**Affiliations:** Department of General, Visceral and Transplantation Surgery, University of Heidelberg, Im Neuenheimer Feld 110, 69120 Heidelberg, Germany; The Study Center of the German Surgical Society (SDGC), University of Heidelberg, Im Neuenheimer Feld 110, 69120 Heidelberg, Germany

**Keywords:** Blinding, Bias, Performance bias, Detection bias, Risk of bias, General and abdominal surgery, Randomized controlled trial, Systematic review

## Abstract

**Background:**

Blinding is a measure in randomized controlled trials (RCT) to reduce detection and performance bias. There is evidence that lack of blinding leads to overestimated treatment effects. Because of the physical component of interventions, blinding is not easily applicable in surgical trials. This is a protocol for a systematic review and empirical study about actual impact on outcomes and future potential of blinding in general and abdominal surgery RCT.

**Methods/design:**

A systematic literature search in CENTRAL, MEDLINE and Web of Science will be conducted to locate RCT between 1996 and 2015 with a surgical intervention. General study characteristics and information on blinding methods will be extracted. The risk of performance and detection bias will be rated as low, unclear or high according to the Cochrane Collaboration’s tool for assessing risk of bias. The main outcome of interest will be the association of a high risk of performance or detection bias with significant trial results and will be tested at a level of significance of 5 %. Further, trials will be meta-analysed in a Mantel-Haenszel model comparing trials with high risk of bias to other trials at a level of significance of 5 %.

**Discussion:**

Detection and performance bias distort treatment effects. The degree of such bias in general and abdominal surgery is unknown. Evidence on influence of missing blinding would improve critical appraisal and conduct of general and abdominal surgery RCT.

**Systematic review registration:**

PROSPERO 2015:CRD42015026837.

**Electronic supplementary material:**

The online version of this article (doi:10.1186/s13643-016-0226-4) contains supplementary material, which is available to authorized users.

## Background

The aim of evidence-based medicine is to find the optimal treatment for patients. This process is based on the expertise of the treating practitioner, the characteristics of patients and the best available external evidence [1]. If available, the best external evidence is data from proper conducted randomized controlled trials (RCT) [2]. Properly conducted RCT take several measures to minimize bias in order to get valid conclusions [3].

Bias describes a systematic error which leads to deviation of the measured effect away from the true effect of an intervention [4]. The Cochrane Collaboration defined the following standard domains of bias: random sequence generation, allocation sequence concealment, blinding of participants and personnel, blinding of outcome assessment, incomplete outcome data, selective outcome reporting and others. These domains are part of critical appraisal in order to judge validity of trials. Influence of bias on quantitative results can be revealed when conducting sensitivity analyses [4]. The CONSORT statement (Consolidated Standards of Reporting Trials), a guideline on reporting of outcomes in randomized controlled trials, declared the information to evaluate these domains of bias as mandatory [5].

One measure to reduce bias is blinding. The risk that awareness of the applied intervention bias effects is called performance bias. Blinding of participants and personnel reduces performance bias. A patient or practitioner who trusts in the effect of a specific intervention may unconsciously or intentionally perceive or detect an enhanced treatment effect [4]. The common term “double-blinded” refers to full avoidance of performance bias by blinding both participants and personnel [6]. Detection bias refers to the risk of how the evaluation of the outcome bias effects. Blinding of outcome assessors reduces detection bias. Outcome assessors (study nurses or investigators) who are aware of the actual treatment may unconsciously or intentionally alter their assessment. Particularly, in case of soft endpoints, e.g. pain blinding of outcome assessors is important. For hard comparators like mortality detection bias is irrelevant [4, 7].

To express bias quantitatively, the association of lack of blinding and significant results is expressed as an odds ratio. From other medical disciplines, four empirical studies [8–11] exist on this topic. Each of them compared the results of clinical trials with absent versus present blinding. A meta-analysis of these empirical studies showed an odds ratio of 0.86 (95 % confidence interval 0.74 to 0.99) demonstrating that lack of blinding leads to overestimated treatment effects [7]. Similarly, the degree of detection bias has been investigated. Blinded and unblinded neurologists assessed a medical intervention to treat multiple sclerosis. Although, no treatment benefit was present, the unblinded neurologists’ scores demonstrated an apparent treatment benefit whereas the blinded neurologists’ scores did not [12].

Due to the physical component of interventions, surgical RCT methodology has some specifics. Blinding of the operating surgeon is sometimes impossible. Blinding of patients and outcome assessors is not easily applicable [3, 4, 13]. For several non-pharmacological treatments, different blinding methods have been investigated, providing detailed methodological information about possible extent of blinding in surgery [14]. However, it remains unclear if ornate blinding measures for surgical interventions are really justified by the gain of better evidence. Cancelling out the blinding measures of patients due to the physical component is very probable and has never been systematically investigated.

Until today, influence of detection and performance bias in general and abdominal surgery RCT is unexplored. The objective of the planned systematic review and empirical study is to investigate actual impact on outcomes and future potential of blinding in general and abdominal surgery RCT.

## Methods/design

This protocol has been prospectively registered under PROSPERO 2015:CRD42015026837 and is written according to the PRISMA-P statement [15] which is included as Additional file 1: PRISMA-P statement.

### Research question

This study aims to determine the status, potential and influence of blinding on outcomes in general and abdominal surgery RCT.

The main outcome is the association of a high risk of detection or performance bias with positive results in general and abdominal surgery RCT.

Secondary outcomes are firstly, the difference of outcomes comparing trials with high risk of bias to trials with low or unclear risk of bias. Secondly, the present status of blinding in general and abdominal surgery will be evaluated by quantification of blinding measures and their reporting in RCT since 1996. Thirdly, the potential of blinding in surgical RCT determined as comparison between possible and actual used blinding methods.

### Systematic literature search

**Fig. 1:**
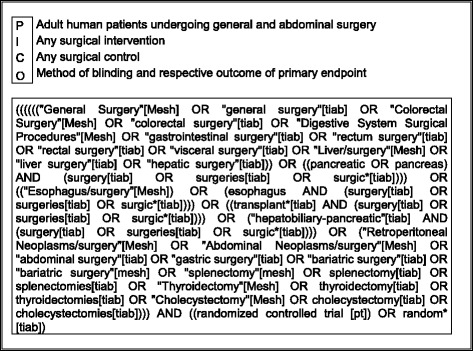
PICO question and search strategy for MEDLINE (via PubMed)

A research question was formulated according to the PICO model (participants, interventions, comparisons and outcomes) [4]. Figure 1 shows the PICO question with the search strategy for MEDLINE (via PubMed). The full search strategy is shown in Additional file 2: Search strategy. The following databases will be searched: CENTRAL, MEDLINE (via PubMed) and Web of Science. A search strategy based on vocabulary thesaurus (MeSH or Emtree) in combination with text words will be used. The search will be limited starting August 1996 when the first CONSORT statement [16] was published and December 2015. No language restrictions will be applied.

### Study selection

Abstract and full-text screening will be performed independently by two reviewers following the recommendations of the Cochrane Collaboration [4]. Articles gathered by the systematic literature search will be screened for eligibility. Randomized controlled trials from general and abdominal surgery with a surgical (non-pharmacological intervention) in adult human patients will be included into full-text screening. A surgical intervention is characterized by a skin incision and a dominant physical component and/or change of anatomy. Good examples for surgical trials to be investigated are comparisons of two surgical accesses (e.g. open vs. laparoscopic), strategies (e.g. Bassini vs. Shouldice in groin surgery) or two possibilities for a specific resection (e.g. hand vs. stapler anastomosis). Trials that investigate surgical education, compare a surgical intervention with a non-surgical intervention (e.g. pharmacologic or radiation) and compare early vs. late time point for operation or more than two study arms will be excluded.

A full-text screening will be performed for all articles eligible after abstract screening. All trials will be checked for proper randomization and allocation according to the recommendations of the Cochrane Collaboration [4]. Moreover, RCT with an a priori defined primary endpoint and an intention-to-treat (ITT) analysis or without unexplained dropout will be included into quantitative analysis.

Including only trials with proper randomization and allocation prevents a high risk of selection bias [4]. An a priori defined primary endpoint is necessary in order to have a sample in which trials are excluded with changed, newly introduced or omitted primary endpoints which therefore are at high risk of bias due to selective reporting [17]. An endpoint is defined a priori if there is an open accessible protocol defining the endpoint as reported or if the primary endpoint is based on a sample size calculation. Including only trials with ITT analysis (with less than 10 % imputated data) or without unexplained dropout prevents high risk of bias due to incomplete outcome data (attrition bias) [4].

**Fig. 2:**
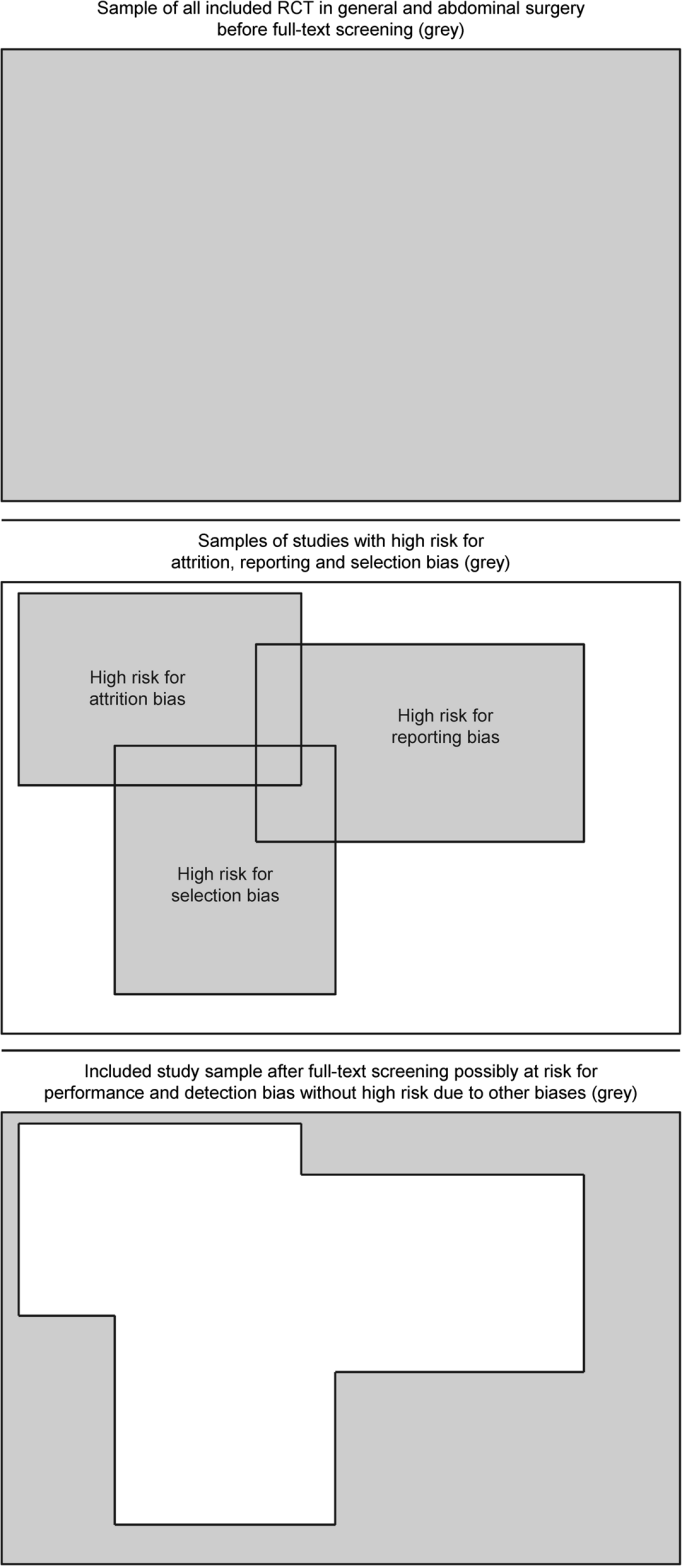
Conceptual visualization of the included study sample

The rationale of the applied eligibility criteria is to achieve a homogenous sample of trials set apart preferably by one point, i.e. the investigated effect of detection and performance bias (Fig. 2).

### Data extraction

The following study characteristics will be extracted: title, author, year of publication, journal, surgical speciality (upper gastrointestinal surgery, hepato-pancreatico-biliary surgery, colorectal surgery/proctology, endocrine surgery, hernia, mixed, others), intervention and control intervention, primary endpoint and outcome. Details of blinding will be extracted from the publication of trials or published protocols belonging to the trials: position in the article where blinding is mentioned (title, abstract, methods section or separate protocol), presence, absence or non-reporting of blinding of trial contributors (patients, practitioners, data collectors, outcome assessors, data analysts), feasibility of blinding trial contributors, risk of performance and detection bias (low, unclear, high), if the influence of missing blinding was discussed and if possible unblinding was assessed during the trial. Funding source is also extracted as another potential source of bias [18]. The original data extraction sheet is shown in Additional file 3: Data extraction sheet.

Data extraction will be performed by two reviewers independently for quality assurance purposes [19]. Discrepancies between the two reviewers will be resolved by a third reviewer, and a final extraction sheet will be determined for database entry. After the last extraction sheet is entered into the database, it will be closed and made available for statistical analysis.

### Data synthesis for the main outcome

Trials will be dichotomized whether they have a high risk of bias due to missing blinding measures or not (low and unclear risk of bias). This is considered to be present if there is a high risk for detection bias or performance bias. Risk for performance and detection bias will be graded as low, unclear or high according to the Cochrane Collaboration’s tool for assessing risk of bias [4]. As mentioned above, blinding is not always possible in surgical trials. As an aid for judgement about feasibility of blinding trial contributors, the systematic review of Boutron et al. will be used [14].

Further, trials will be dichotomized whether they have a significant result or not at a level of significance of <5 %.

### Statistical analysis

Primary statistical analysis and meta-analysis will be performed with program R [20] and Revman 5.3.5 [21], respectively.

#### Main outcome

The null hypothesis (H0) is that a high risk of performance or detection bias is not associated with significant results. The alternative hypothesis (H1) is that a high risk of performance or detection bias is associated with significant results.

The main outcome will be evaluated for performance and detection bias separately. A test at a level of significance of 5 % on the association of high risk of bias and significant results will be performed.

The significance of association will be tested by means of Fisher’s exact test if at least one value in the contingency table is 5 or below. Pearson’s chi-squared test with Yates’s correction will be used if the total sample size is 60 or less. In all other cases, significance of association will be tested using Pearson’s chi-squared test without Yates’s correction [22].

#### Secondary outcomes

A separate meta-analysis for binary and continuous data will be conducted with random-effects Mantel-Haenszel model comparing the high risk of bias trials with the rest of trials at a level of significance of 5 % for subgroup difference. Publication bias will be explored using a funnel plot separately for trials at high risk and not at high risk.

The rate of blinded trials and respective blinded study contributors will be expressed descriptively overall and over time periods (1996–2000, 2001–2009, 2010–2015).

The potential of blinding will be expressed as comparison of actual blinded trials with feasibility of blinding in included trials.

#### Subgroup and sensitivity analysis

For the main outcome and secondary outcomes, subgroup analyses will be performed according to which of the study contributors were blinded. Additionally, a subgroup analysis will be performed according to the intervention type in included trials, i.e. investigating operative accesses, different instruments or surgical strategies.

A sensitivity analysis of the main outcome will be performed excluding all trials with an objective primary endpoint, e.g. mortality. Furthermore, a sensitivity analysis for the main outcome will be performed comparing trials at low risk of bias with high/unclear risk of bias and comparing trials at high risk of bias with trials at low risk of bias only.

## Discussion

This protocol describes the methods of a systematic review and empirical analysis, which will provide the present status and potential and influence of blinding on outcomes in general and abdominal surgery RCT.

The study sample included for analysis will be specific for general and abdominal surgical RCT. To narrow the analysis on the influence of detection and performance bias, other sources of biases are excluded. In the preliminary search, about 30,000 articles were found. With the above described eligibility criteria, an estimated precision of 3 % and other acquainted values from a former review [18, 22], a sample of about 600 RCT will be expected to be available for quantitative analysis.

The existing literature clearly shows that existing detection and performance bias distort the measured effect from the true effect in pharmacological trials [7, 12]. In contrast to a pill, surgical interventions cannot be blinded to all trial contributors. The degree of such bias in general and abdominal surgery has never been determined quantitatively. Therefore, the conduct of this study is important because the knowledge about the influence of missing blinding could further be discussed not only in a qualitative but also in a quantitative manner. By using the established Cochrane Collaboration’s tool for assessing risk of bias [4], the results of the planned analysis will be specifically applicable to critical appraisal of surgical trials. Possible overestimated treatment effects could be detected and corrected or at least be discussed on quantitative evidence. In case of a missing association between lack of blinding and significant trial results, different reasons have to be taken into account. One reason would be publication bias with due to negative and assumably blinded trials which were restrained. Another reason could in fact be the strong physical component of surgical interventions leading to unblinding of patients. This reason can be explored if trials evaluated the unblinding rate of their measures. However, it is also possible that in surgical trials, blinding has not the same status than in pharmacological trials. In case of a true missing association between lack of blinding and significant trial results, surgical researcher could rely on this evidence and leave out complicated ways of blinding methods in RCT without threatening the validity of trial results.

## Additional files

Additional file 1: PRISMA-P statement. (DOC 82 kb) Additional file 2: Search strategy. (DOCX 16 kb) Additional file 3: Data extraction sheet. (DOCX 27 kb)

## Abbreviations

ITT: intention to treat
PICO: participants, interventions, comparisons and outcomes
RCT: randomized controlled trials
