# Supplementary material for: Blinding in randomized controlled trials in general and abdominal surgery: protocol for a systematic review and empirical study
Source: Syst Rev. 2016 Mar 24;5:48. doi: 10.1186/s13643-016-0226-4 (PMC4806514; doi:10.1186/s13643-016-0226-4)
Supplement: Additional file 2: — Search strategy. (DOCX 16 kb) [file 13643_2016_226_MOESM2_ESM.docx]

**Search strategy**

**MEDLINE (via PubMed)**

(((((("General Surgery"[Mesh] OR "general surgery"[tiab] OR "Colorectal Surgery"[Mesh] OR "colorectal surgery"[tiab] OR "Digestive System Surgical Procedures"[Mesh] OR "gastrointestinal surgery"[tiab] OR "rectum surgery"[tiab] OR "rectal surgery"[tiab] OR "visceral surgery"[tiab] OR "Liver/surgery"[Mesh] OR "liver surgery"[tiab] OR "hepatic surgery"[tiab])) OR ((pancreatic OR pancreas) AND (surgery[tiab] OR surgeries[tiab] OR surgic*[tiab]))) OR (("Esophagus/surgery"[Mesh]) OR (esophagus AND (surgery[tiab] OR surgeries[tiab] OR surgic*[tiab]))) OR ((transplant*[tiab] AND (surgery[tiab] OR surgeries[tiab] OR surgic*[tiab]))) OR ("hepatobiliary-pancreatic"[tiab] AND (surgery[tiab] OR surgeries[tiab] OR surgic*[tiab]))) OR ("Retroperitoneal Neoplasms/surgery"[Mesh] OR "Abdominal Neoplasms/surgery"[Mesh] OR "abdominal surgery"[tiab] OR "gastric surgery"[tiab] OR "bariatric surgery"[tiab] OR "bariatric surgery"[mesh] OR "splenectomy"[mesh] OR splenectomy[tiab] OR splenectomies[tiab] OR "Thyroidectomy"[Mesh] OR thyroidectomy[tiab] OR thyroidectomies[tiab] OR "Cholecystectomy"[Mesh] OR cholecystectomy[tiab] OR cholecystectomies[tiab]))) AND ((randomized controlled trial [pt]) OR random* [tiab])

**CENTRAL**

ID           Search

#1           "general surgery":ti,ab,kw  (Word variations have been searched)

#2           MeSH descriptor: [General Surgery] explode all trees

#3           #1 or #2

#4           "colorectal surgery":ti,ab,kw  (Word variations have been searched)

#5           MeSH descriptor: [Colorectal Surgery] explode all trees

#6           #4 or #5

#7           "gastrointestinal surgery":ti,ab,kw  (Word variations have been searched)

#8           "rectum surgery" or "rectal surgery " or "visceral surgery":ti,ab,kw  (Word variations have been searched)

#9           #7 or #8

#10        "liver surgery":ti,ab,kw  (Word variations have been searched)

#11        "hepatic surgery":ti,ab,kw  (Word variations have been searched)

#12        "pancreas surgery" or "pancreatic surgery":ti,ab,kw  (Word variations have been searched)

#13        "esophagus surgery" or "oesophagus surgery":ti,ab,kw  (Word variations have been searched)

#14        "transplant surgery" or "transplantation surgery":ti,ab,kw  (Word variations have been searched)

#15        hepatobiliary-pancreatic surgery:ti,ab,kw  (Word variations have been searched)

#16        "abdominal surgery" or "gastric surgery" or "bariatric surgery" or splenectomy:ti,ab,kw  (Word variations have been searched)

#17        MeSH descriptor: [Bariatric Surgery] explode all trees

#18        MeSH descriptor: [Splenectomy] explode all trees

#19        #10 or #11 or #12 or #13 or #14 or #15 or #16 or #17 or #18

#20        thyroidectomy:ti,ab,kw  (Word variations have been searched)

#21        MeSH descriptor: [Thyroidectomy] explode all trees

#22        #20 or #21

#23        MeSH descriptor: [Cholecystectomy] explode all trees

#24        cholecystectomy:ti,ab,kw  (Word variations have been searched)

#25        #23 or #24

#26        pediatric or child or animal or pig or rat:ti,ab,kw  (Word variations have been searched)

#27        letter or comment or editorial:ti,ab,kw  (Word variations have been searched)

#28        #3 or #6 or #9 or #19 or #22 or #25

#29        #28 not 26

#30        #29 not #27 Publication Year from 1996 to 2015

**Web of Science**

#6 #2 AND #5

DocType=All document types; Language=All languages;

#5 #4 OR #3

DocType=All document types; Language=All languages;

#4 ti=(clinical near trial* or crossover or cross over or placebo* or random*)

DocType=All document types; Language=All languages;

#3 ts=(clinical near trial* or crossover or cross over or placebo* or random*)

DocType=All document types; Language=All languages;

#2 TS=("general surgery" OR "colorectal surgery" OR "gastrointestinal surgery" OR "rectum surgery" OR "rectal surgery" OR "visceral surgery" OR "liver surgery" OR "hepatic surgery" OR "pancreatic surgery "OR "esophagus surgery" OR "transplantation surgery" OR "abdominal surgery" OR "gastric surgery" OR "bariatric surgery" OR splenectomy OR splenectomies OR thyroidectomy OR thyroidectomies OR cholecystectomy OR cholecystectomies)

DocType=All document types; Language=All languages;

#1 TOPIC: ("general surgery" OR "colorectal surgery" OR "gastrointestinal surgery" OR "rectum surgery" OR "rectal surgery" OR "visceral surgery" OR "liver surgery" OR "hepatic surgery" OR "pancreatic surgery "OR "esophagus surgery" OR "transplantation surgery" OR "abdominal surgery" OR "gastric surgery" OR "bariatric surgery" OR splenectomy OR splenectomies OR thyroidectomy OR thyroidectomies OR cholecystectomy OR cholecystectomies)

DocType=All document types; Language=All languages;
