# Supplementary material for: Blinding in randomized controlled trials in general and abdominal surgery: protocol for a systematic review and empirical study
Source: Syst Rev. 2016 Mar 24;5:48. doi: 10.1186/s13643-016-0226-4 (PMC4806514; doi:10.1186/s13643-016-0226-4)
Supplement: Additional file 3: — Data extraction sheet. (DOCX 27 kb) [file 13643_2016_226_MOESM3_ESM.docx]

**Study-ID:       Data Extractor**:  PP  2nd  DB

**Characteristics**

**Title:**

**First Author:** **Pub Year:** **Journal:**

**Intervention/ Outcome**

**Operation  Upper GI  HPB  Colo-rectal/Procto  Endocrine  Hernia  Mixed  Other:**

**Intervention  Access  Strategy  Instruments  Other:**

**Primary Endpoint:**

**Binary: I:      /      C:      /**

**Continuous: I: Mean      Sd      Pop      C: Mean      Sd      Pop**

**I: Median      Range      Pop      C: Median      Range      Pop**

**Significant:** **Yes**  **No**

**Blinding**

**Blinding mentioned:  Title  Abstract  Methods**

**Protocol available  Not mentioned  Mentioned**

**“blinded”  “single-blinded”  “double-blinded”  “triple-blinded”  Other:**

**Patients****:  Blinded  Not blinded  Not stated**

**Practitioners****:  Blinded  Not blinded  Not stated**

**Data collectors:  Blinded  Not blinded  Not stated**

**Outcome assessors****:  Blinded  Not blinded  Not stated**

**Data analysts:  Blinded  Not blinded  Not stated**

**Feasibility of Blinding?  Patients  Practitioners  Data collectors**

**Outcome assessors  Data analysts**

**Risk of Performance Bias  Low  High  Unclear**

**Risk of Detection Bias  Low  High  Unclear**

**Influence of missing blinding discussed?  Yes:        No**

**Unblinding assessed?  Yes:        No**

**Funding:  Industry  Non-industry  Not stated**

**Important notes:**

**Date:       Signature:**
